# Monomer Trapping Synthesis Toward Dynamic Nanoconfinement Self‐healing Eutectogels for Strain Sensing

**DOI:** 10.1002/advs.202410446

**Published:** 2024-09-16

**Authors:** Yuesong Lv, Changchun Li, Zhangqin Yang, Mingxi Gan, Yuyan Wang, Minxun Lu, Xinxing Zhang, Li Min

**Affiliations:** ^1^ State Key Laboratory of Polymer Materials Engineering Polymer Research Institute of Sichuan University Chengdu 610065 China; ^2^ Orthopedic Research Institute Department of Orthopedics West China Hospital of Sichuan University Chengdu 610065 China; ^3^ Model Worker and Craftsman Talent Innovation Workshop of Sichuan province West China Hospital Sichuan University chengdu 610065 China; ^4^ Max Planck Institute for Polymer Research Ackermannweg 10 55128 Mainz Germany

**Keywords:** dynamic nanoconfinement, eutectogel, monomer trapping synthesis, self‐healing, strain sensing

## Abstract

The rapid advancement in attractive platforms such as biomedicine and human‐machine interaction has generated urgent demands for intelligent materials with high strength, flexibility, and self‐healing capabilities. However, existing self‐healing ability materials are challenged by a trade‐off between high strength, low elastic modulus, and healing ability due to the inherent low strength of noncovalent bonding. Here, drawing inspiration from human fibroblasts, a monomer trapping synthesis strategy is presented based on the dissociation and reconfiguration in amphiphilic ionic restrictors (7000‐times volume monomer trapping) to develop a eutectogel. Benefiting from the nanoconfinement and dynamic interfacial interactions, the molecular chain backbone of the formed confined domains is mechanically reinforced while preserving soft movement capabilities. The resulting eutectogels demonstrate superior mechanical properties (1799% and 2753% higher tensile strength and toughness than pure polymerized deep eutectic solvent), excellent self‐healing efficiency (>90%), low tangential modulus (0.367 MPa during the working stage), and the ability to sensitively monitor human activities. This strategy is poised to offer a new perspective for developing high strength, low modulus, and self‐healing wearable electronics tailored to human body motion.

## Graphical abstract

A dynamic nanoconfinement eutectogel sensor has been fabricated by monomer trapping synthesis based on dissociation and reconfiguration of the “locking” network of amphiphilic ionic restrictors. The composites are endowed with nanoconfinement and dynamic interface to retain low modulus and motion properties. This strategy shows a solution for developing wearable devices adapted to tissue movement in cutting‐edge fields.

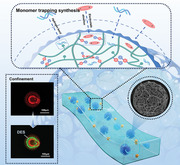

## 1 Introduction

Self‐healing wearable flexible sensors show broad potential applications in biomedicine, human‐machine interfaces, and bionic skin due to their high extensibility and repairable functions.^[^ ^1^, ^2^, ^3^, ^4^ ^]^ Human motion is always complex and diverse, necessitating flexible sensors that possess both adequate tenacity to resist stress and sufficient suppleness to adapt to movements.^[^ ^5^, ^6^ ^]^ It is worth noting that the self‐healing materials typically sacrifice its mechanical robustness because of reversible interactions, and possess a mutually exclusive governing between high strength and low modulus.^[^ ^7^, ^8^, ^9^, ^10^ ^]^ Various molecular engineering techniques, such as tensile training,^[^ ^11^ ^]^ annealing for covalent bonding,^[^ ^12^ ^]^ hybridization networks,^[^ ^13^ ^]^ and crystallization,^[^ ^14^ ^]^ have been employed to bolster the strength and durability. However, these methods unintentionally “harden” the material, compromising its self‐healing ability and elevating its modulus. Achieving a balance between robustness and low modulus that approximates to soft tissue movements in a repairable system has been rarely documented in current literature.

Recently, nanoconfinement materials inspired by microscopic living systems have emerged as strong candidates for robustness and self‐healing ability.^[^ ^15^, ^16^, ^17^, ^18^ ^]^ Nanoconfinement techniques impose semi‐dynamic confinement loading by small‐dimensional restrictors to hinder molecular chain movement and slip.^[^ ^19^ ^]^ Song et al. developed a super‐tough material by incorporating small multiamine molecules as hydrogen‐bonding crosslinkers, resulting in multiple hydrogen‐bonding dynamic confined domains within PVA.^[^ ^20^ ^]^ Yan et al. constrained the polymerization of polyacrylamide within hydrogen‐bond‐rich COFs or molecular sieves, creating a confined phase that acts as a stress transfer and dissipation center.^[^ ^21^ ^]^ Unfortunately, while cleverly circumvent the weakening effect of reversible interactions from a mechanical point of view, the rigid microdomains limit the deformation of the flexible material and result in consistent trends in strength and modulus.^[^ ^22^, ^23^, ^24^ ^]^ The challenge of balancing low modulus and high strength remains unresolved.

The loose connective tissues (LCTs) connecting the organ with bone coordinate the movement of the protein fiber network by its fibroblasts serving as intrinsic repairable and soft restrictors.^[^ ^25^, ^26^ ^]^ While the substrates are enhanced by confinement effect, the soft confined domains still have excellent movement properties, allowing for sufficient relaxation space and a smooth molecular motion process. Moreover, the self‐repairing mechanism grounded on the inherent self‐healing capacity of cells, is naturally integrated into the dynamic equilibrium of connective tissues. This provides inspiration for the preparation of robust, flexible polymers fitting tissue movement.

Here, we propose a dynamic nanoconfinement strategy based on monomer trapping synthesis to achieve low modulus, high strength, and self‐healing simultaneously. Amphiphilic ionic microspheres (ADMs), which trap polymerizable deep eutectic solvents (PDES) in excess of 7000 times volume by dissociating and reconfiguring their own self‐associated network, build dynamically bonded confined domains. Besides, the soft PDES network interpenetrates with the restrictor backbone similarly to LCTs and thus reserves soft deformation capability. The developed eutectogel perfectly integrates the seemingly contradictory properties such as robustness, low modulus, and hard‐soft‐hard deformation style, while exhibiting excellent self‐healing ability and electron conduction. This study offers valuable insights for the advancement of robust and self‐healing soft sensors.

## 2 Results and Discussions

### 2.1 Monomer Trapping Synthesis of Nanoconfinement ADMs

**Figure 1:**
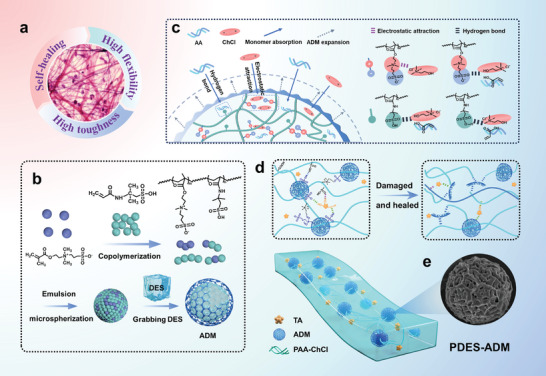
Materials design of dynamic nanoconfinement eutectogel sensors. a) Staining optical pictures and properties of loose connective tissues (LCTs) in human organs. b) Schematic illustration of the ADM synthesis process. c) Schematic representation of ADM monomer trapping and Chemical formulas’ representation of electrostatic attraction and specific multiple hydrogen bonding interactions inducing ADM's trapping of monomers. d) Schematic illustration of the PDES‐ADM structure and its behavior in the presence of multiple hydrogen bonds self‐healing. e) SEM image of ADM.

The human body's LCTs act as a “seatbelt” between organs and muscles, possessing both robustness and self‐repair capabilities (**Figure** **1**a). This pliable biological tissue comprises soft fibroblast nodes enveloping protein chains. The restrictive structure allows it to securely hold organs and bones during human movement. Taking inspiration from LCTs, we plan to disperse the tight confinement structure to attenuate hardening, which allows the confined domains to deform with the movement of the substrate. This distinguishes it from delicate and hard nano‐restrictors. However, it is difficult for polymer chains to build inhomogeneous microstructures under confined space.^[^ ^27^, ^28^, ^29^ ^]^ To address this problem, we build confined domains by monomer trapping synthesis. ADMs were developed to access the nanoconfinement capabilities of LCTs by the copolymerization of sulfonic acid‐based monomers (AMPS) and sulfobetaine (DMAPS) (Figure 1b). Through strong solvation effects and hydrogen bonding active sites conferred by sulfonate functional groups, ADMs ultimately trapping mobile deep eutectic solvent (DES) more than 7000 times their own volume with the aid of tannic acid (TA) (Figure 1c; Figure S1, Supporting Information).^[^ ^30^, ^31^ ^]^ Subsequently, the confinement domains are preserved by the polymerization of DES (Figure S10, Supporting Information). The inherent microphase separation of the confined hardened ADMs backbone from the abundant soft molecular chains of PDES promotes the soft properties of the confined domains. ADMs surfaces and sphere matrices are rich in highly polar sulfonic acid groups and dynamic electrically coupled amphiphilic ions structures, which form highly reactive hydrogen bonds with dynamic intermolecular electrostatic interactions with the side groups of the PAA molecular chain. This confers the confined domains the dynamic ability to re‐establish supramolecular interactions through molecular chain relaxation motions when the material is disrupted by external forces (Figure 1d).

**Figure 2:**
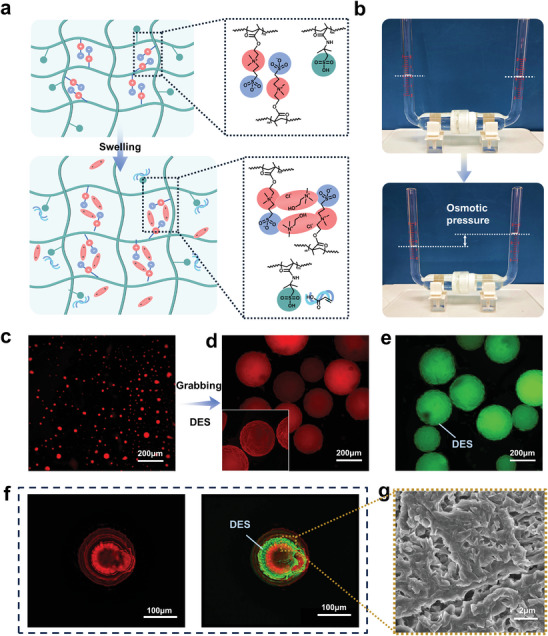
ADM's monomer‐trapping confinement effect and characterization of morphology. a) Schematic illustration of the structural changes in the molecular chain network with a “locking” effect of ADM before and after reconfiguration. b) Photos of the difference in liquid level before and after the addition of ADM microspheres in a U‐shaped simplified osmosis device (≈1.6 ml volume difference). Laser confocal microscopy shots of c) unswollen ADM in isopropanol, d) swollen ADM in DES (inset is a 3D scanned image), and e) green channel monomer signals under ADM confinement. f) 3D reconstructed image of the ADM scanning cross‐section, the green part represents the confined DES. g) SEM image of cross‐section of expanded ADM.

The ability of ADMs to trap monomers is attributed to amphiphilic side groups with self‐association properties in the network structure.^[^ ^32^ ^]^ As shown in **Figure** **2**a, the presence of positively and negatively charged ions attached on molecular chains creates a strong “locking” effect, resulting in a tightly closed network.^[^ ^33^, ^34^ ^]^ Introduction of ionic liquid like DES causes the free active choline chloride (ChCl) to seek electrical equilibrium, leading to the unlocking of the network and binding with ChCl. Furthermore, the abundant sulfonic acid side groups form hydrogen bonds with the active hydrogen bonding proton donor acrylic acid (AA). These characteristics allow ADMs to engage in various electrostatic and hydrogen bonding interactions with DES, effectively establishing homogeneous confined monomer regions. The adsorption of ADMs onto solvents was confirmed using a simplified osmotic pressure apparatus. As shown in Figure 2b, the introduction of ADMs on the right side of a U‐tube with a semi‐permeable membrane barrier intuitively produces a difference in volume height of ≈1.6 ml after 12 h for liquid level stabilization. This outcome qualitatively indicates that there is a strong interaction between ADM and the solvent, generating a solvation osmotic pressure.

To visually confirm the monomer trapping process, the confinement effect of microspheres was characterized using laser confocal microscopy.^[^ ^35^ ^]^ The initial volume‐averaged particle size of the ADMs is ≈13.4 µm (Figure 2c; Figure S2, Supporting Information). Upon introduction into the DES solvent, ADMs entrap the liquid monomer, leading to volume expansion (with an average particle size of ≈259.5 µm after 12 h) (Figure 2d; Figure S3, Supporting Information). It is calculated that more than 7000 times of the starting volume of monomers are confined under strong interaction forces. Simultaneously, ADMs exhibit uniform green channel monomer signals (Figure 2e). This confirms that the reorganization of the amphiphilic ion self‐locked network manifests itself macroscopically as a volume expansion of the ADMs. To explicitly confirm the morphology of the confined monomer, 3D reconstructions of cross‐sectional scans of the cured microspheres were conducted. The DES presents a flower cluster shape and is situated within the ADMs as shown in Figure 2f, illustrating the successful construction of the confinement effect. In addition, an SEM image of the cross‐section of the swollen ADMs post freeze‐drying (removal of confined monomers) reveals the presence of numerous nanoscale spatial grooves (Figure 2g; Figure S4, Supporting Information). This provides lateral evidence of microphase separation in the confined domain space between the hardened backbone of the ADMs and the soft PDES chains. Further, if a long period of time was reserved for ADM for monomer capture, the sphere particle size still continued to rise until it stabilized after 7 days. As shown in Figure S9 (Supporting Information), the particle size of the spheres swelled to 532.72 µm at 10 days. However, compared to the decreased performance of PDES‐ADM with 12 h monomer capture polymerization. Thus, 12 h monomer capture polymerization is still taken as the main research objective in this paper.

### 2.2 Reversible Interfacial Hydrogen Bonding Networks

**Figure 3:**
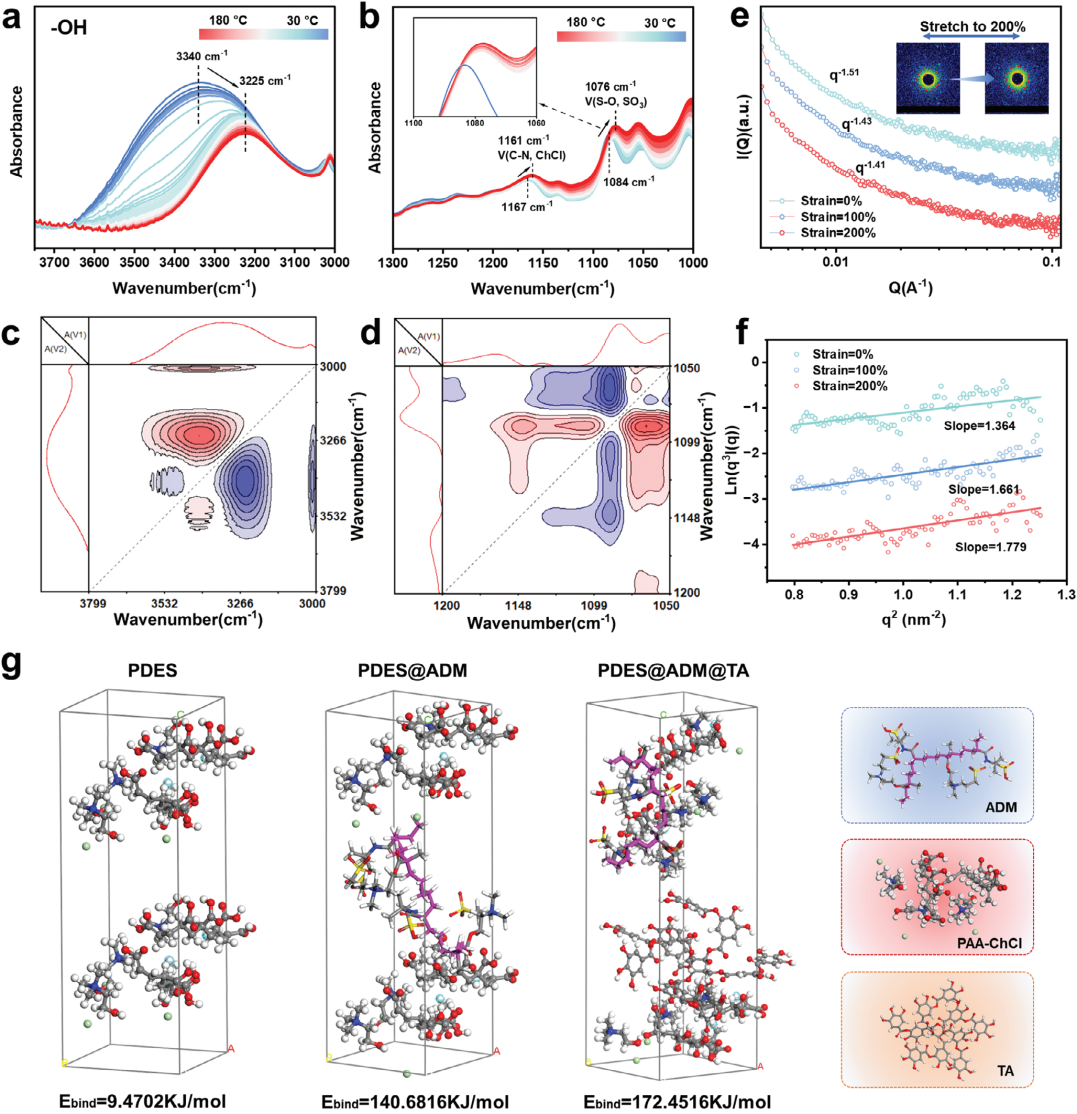
Characterizations of multiple hydrogen bonding at interfaces between ADM and eutectogel. Temperature‐variable FTIR spectra of the PDES‐ADM upon heating from 30 to 180 °C in the range of a) 3750–3000 and b) 1300–1000 cm^–1^. Generalized 2D correlation spectra of the PDES‐ADM upon heating from 30 to 180 °C in the range of c) 3800–3000 and d) 1200–1050 cm^−1^. e) 1D scattering profiles of the PDES‐ADM composites under strains of 0, 100, and 200%. Inset: 2D SAXS pattern obtained during uniaxial stretching. f) SAXS plots of ln(q^3^I(q)) versus q^2^ for PDES‐ADM at different strains. g) Optimized structures and binding energies of PDES, PDES@ADMS and PDES@ADM@TA.

The self‐conjugated network of ADM restrictors rebuilds interactions with the monomer, which ensures a highly compatible system dispersed between the restrictors. Subsequently, polymerization initiation converts the monomers into polymers, and the resulting eutectogels retain the interfacial interactions between the molecular chains and the restrictor structures, resulting in the construction of dynamic nanoconfinement characterized by dynamic bonding. In order to probe the multiple hydrogen bonding behaviors in PDES‐ADM, temperature‐variable FTIR spectroscopy from 30 to 180 °C in conjunction with generalized 2D correlation spectroscopy (2DCS) was used to analyze the interactions within the dynamic nanoconfinement eutectogels (**Figure** **3**a–d; Figure S5 Supporting Information).^[^ ^36^, ^37^ ^]^ With the temperature increasing, a significant red‐shift from 3340 to 3225 cm^−1^ peak is observed belonging to the ─OH groups. Red shift is also observed at peaks 1084 and 1167 cm^−1^ belonging to ─SO_3_ and ChCl, moving to 1076 and 1161 cm^−1^ respectively. In contrast, the 1725 cm^−1^ peak belonging to ─C═O is blue‐shifted to 1730 cm^−1^. The red shift of the ─OH group is typical of the hydrogen bond formation process in which ─OH is transformed from the disassociated to the associated type, and the red shift of ChCl also represents part of the hydrogen bond formation associated with it. Irregularly, the red‐shift of the ─SO_3_ group band in the IR spectra represents the formation of isolated ─SO_3_^−^, which implies the dissociation of ─SO_3_ hydrogen bonds.^[^ ^38^ ^]^ Also, the blue‐shifted band of ─C═O represents the corresponding hydrogen bond dissociation. This suggests that ─SO_3_ like hydrogen bonds belonging to ADMs weaken the ─OH hydrogen bonding and ─C─N hydrogen bonding inherent in PDES, and tend to establish more robust interacting hydrogen bonds between ADMs and PDES at room temperature. At the same time the observations of ascending changes in the peaks of the spectral bands belonging to ChCl and ─SO_3_^−^ with increasing temperature represent an intensification of the ionic activity associated with them, indicating an increase in the electrical interaction of ADMs with ChCl. The results above show that multiple noncovalent interactions in the systems are highly active, which lays a foundation for energy dissipation of nanoconfined domains under external forces.^[^ ^39^ ^]^

Interface interactions can synergize with nanoconfinement effects to alter the motion behavior of molecular chains in confined areas at the microscopic level. In situ, Small‐angle X‐ray scattering (SAXS) analysis was conducted on PDES‐ADM to demonstrate the impact of supramolecular confined domains on the material's strain behavior. As strain increases, the scattering signal transitions from an isotropic circular shape to a directional elliptical shape, rather than adopting a rhombus form. This indicates that the confinement effect stabilizes the longitudinal structure of the confined domain during orientation (Figure 3e). The scattering curve of the low Q segment (Q < 0.01) from 1D SAXS shifts from q^−1.51^ to q^−1.41^, suggesting that the sacrificial dissociation of dynamic bonds during the stretching process expands the molecular distance, leading to a decrease in the electron cloud density of the eutectogel molecular chain. Furthermore, the synergistic effect of nanoconfinement and interfacial interaction is investigated by comparing the thickness of the interface layer. The thickness of the interface layer can be defined from the SAXS results, in accordance with Porod's law.

(1) $$E\;=\sqrt{2\pi \lambda }$$

where E is the thickness of the interfacial layer and λ is the slope of Ln (q^3^ *I*(q)) versus q^2^ in the high q range. The measured value of λ at 0% and 200% tensile strain increases from 1.364 to 1.779, which indicates that the thickness of the interfacial layer grows from 2.928 to 3.343 nm (Figure 3f). This increase of the interfacial layer during the tensile process is likely attributed to the protective role of the confinement effect, which preserves the structural stability and thereby enables the continuous generation of new interfacial layers through the highly active dynamic bonding. This microscopically avoids physical disruption between the polymer matrix and the restriction domains.

The binding energies between the PDES (PAA/ChCl) matrix and ADMs fillers (TA modified or unmodified) were calculated by isothermal‐isotropic molecular dynamics (MD) simulations, and the positive effects of the introduction of ADMs and TA on the establishment of interfacial secondary interactions (multiple hydrogen bonding) were investigated.^[^ ^40^, ^41^ ^]^ We randomly combined PAA and ChCl by adsorption modules into a simplified ion‐elastomer matrix, and subsequently, we simplified a single ADMs molecular chain into two repetitive units and constructed PDES cells, TA cells, and PDES and ADMs molecular chains with the same volume ratio in an amorphous crystalline cell system (PDES@ADM) cell. Geometrical optimization was performed for each cell, and then PDES@ADM@PDES was obtained by building layers with PDES as layer 1 and PDES@ADM as layer 2. Dynamical optimization was performed at 298 K under a generic force field of 1000 PS to obtain the optimal conformation, and the resulting molecular model was used to calculate the potential energy of the whole system (E_total_). Then, the corresponding energy of PDES (E_substrate_) or ADM (E_filler_) was calculated by removing PDES or ADMs without any other changes. The binding energy can be calculated according to the following equation:

(2) $$E_{\mathrm{bind}}\;=\;E_{\mathrm{substrate}}+E_{\mathrm{filler}}-E_{\mathrm{total}}$$

where E_bind_ is the binding energy of the polymer to the filler; E_substrate_ and E_filler_ denote the corresponding energies of the polymer matrix and filler in the optimized conformation, and E_total_ is the total energy of the system. The binding energy of the PDES system was obtained by the same operation without the introduction of the ADMs. The binding energy of the TA‐modified PDES@ADM@PDES system was also obtained by the same operation, and their modeled molecular dynamics simulations are shown in Figure 3g, respectively. The introduction of ADMs into the PDES system changes the calculated value of the binding energy from 9.47 to 140.68 kJ mol^−1^, which increases 13.85 times. The enhanced interfacial interactions reflected by the binding energy are attributed to the multiple hydrogen bonding and electrostatic attraction of the two kinds of sulfonated side groups of ADMs to DES, which constructs a dense and strong non‐covalent crosslinked network. In addition, TA acts as an interfacial hydrogen bonding infiltrant to improve the degree of hydrogen bonding match between the two, further increasing the binding energy to 172.45 kJ mol^−1^.

### 2.3 Mechanical and Self‐Healing Properties

**Figure 4:**
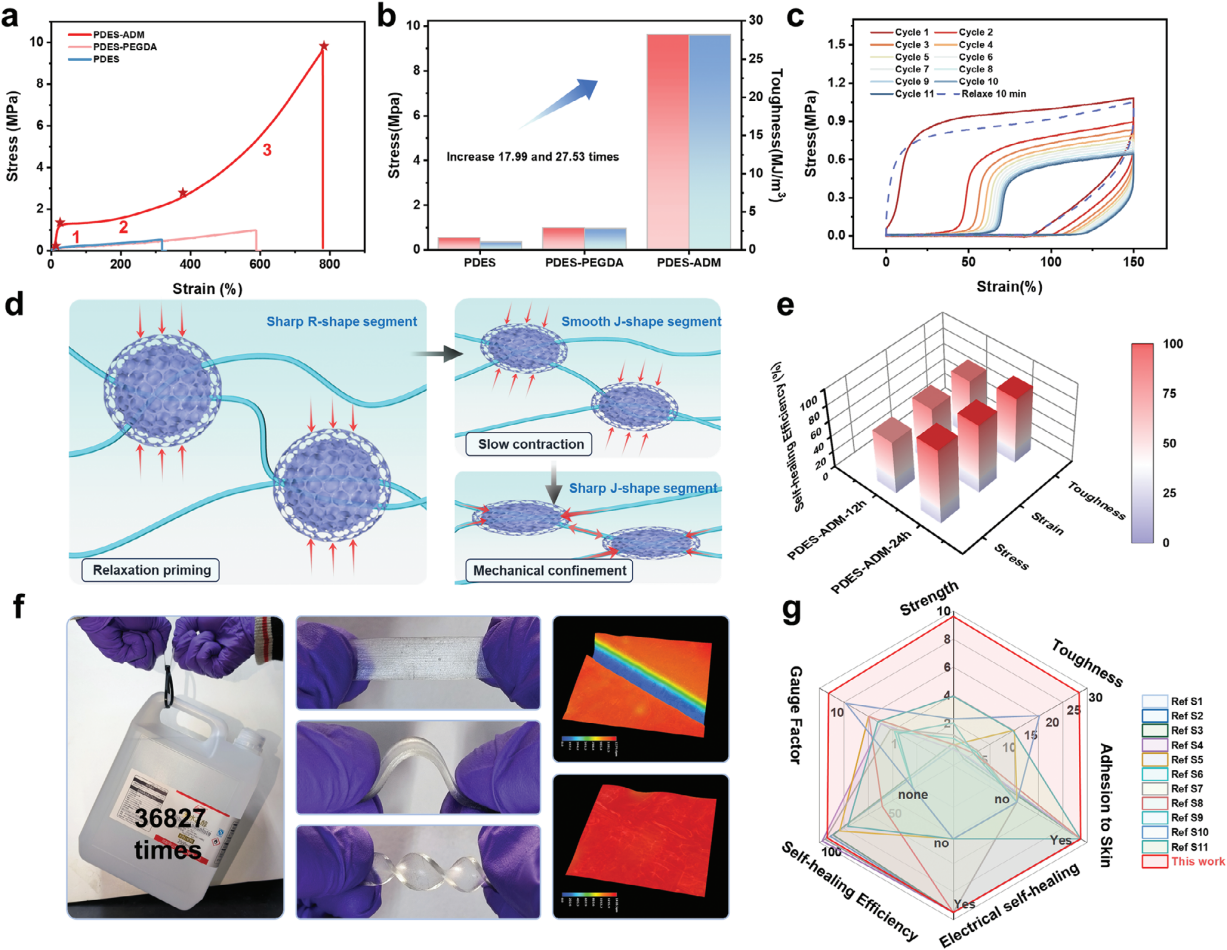
Mechanical properties and self‐healing. a) Stress–strain curves of PDES‐ADM, PDES‐PEGDA, and PDES under tension. Asterisked points are stress‐strain pattern change points. b) Comparison of tensile strength and toughness of PDES–ADM, PDES‐PEGDA, and PDES. c) Cyclic loading and recovery of PDES‐ADM. The samples were conducted cyclic loading for eleven times (150 % strain) at a tensile rate of 50 mm min^−1^, and then the samples were relaxed for 10 min at room temperature to release the strain and stretch again. d) Schematic diagram of the movement of the confined domain during stretching e) Self‐healing efficiency of maximum tensile strength, strain, and fracture toughness of PDES‐ADM at different self‐healing times. f) Photos of healed samples stretching, bending, and twisting, and the healed sample (weight: 0.068 g) lifting 36 827 times (2500 g) its own weight. Ultra‐depth‐of‐field 3D microscopy images of cut and healed PDES‐ADM samples. g) Comparison of ultimate tensile strength, toughness, self‐healing efficiency, adhesion ability, GF values, and Electrical self‐healing of our PDES‐ADM with other flexible ion sensors.

The PDES‐ADM's soft chain segments are tightly interspersed with the ADMs backbone reinforced by dynamic bound in the confined domains. This integration elevates the overall strength and tensile properties to 9.62 MPa and 780.32%, respectively (**Figure** **4**a). Consequently, the PDES‐ADM exhibits a significant enhancement in maximum strength (17.99 times) and toughness (27.53 times) when compared to pure PDES (Figure 4b). Furthermore, the softness confined domains alter the stress change pattern of PDES‐ADM, presenting a non‐conventional hard‐soft‐hard trend. This can be broadly categorized into three stages: sharp R‐shape (segment 1), smooth J‐shape (segment 2), and sharp J‐shape (segment 3, non‐linear strengthening) (Figure 4d). In the R‐shaped state (5.484 MPa tangential modulus), the stress differential curve of PDES‐ADM shows a clear relaxation peak (Figure S6, Supporting Information). The rigid dynamic bonding segments allow for a slower initial relaxation of the confined domains than the molecular chain backbone, resulting in a strong hardening elasticity. This confers PDES‐ADM a resistance effect during the onset of human locomotion. In the smooth J‐shaped state (0.367 MPa tangential modulus), the confined domains undergo gradual deformation, accompanied by weak hydrogen bond dissociation and electrostatic interaction detachment. This state accommodates the vast majority of the body's motion. In the sharp J‐shaped state (1.668 MPa tangential modulus), when the confined domains exhibit a high degree of orientation, great mechanical binding occurs due to the tautness of the chain segments.

To further verify the impact of nanoconfinement effects and interfacial interactions on strain‐induced structural transitions, we investigated the mechanical properties of eutectogels with different degrees of monomer capture synthesis (Figure S8 Supporting Information). A composite that has undergone no time for monomer trapping does not exhibit the R‐shaped platform characterized by nanoconfinement, thus we regard it as a state without confined domains. At this point, the strain capacity of the material is still much higher than that of pure PDES. This is attributed to the dense dynamic interactions between the ADM surface and the external gel that regenerate the interfacial layer while the molecular chains are forced to slide. Eutectogel with a higher degree of monomer trapping has significantly higher fracture stress and maintains high strain. This implies that the confined domains play an important role in strengthening the molecular chain backbone and in synergy with the interfacial interactions to enhance the dynamic stability of the structure (in agreement with the SAXS analysis). The closed domains exhibited a more pronounced effect on the material in cyclic stretching (Figure 4c). Multiple dissociable sulfonyl hydrogen bonds with electrostatic interactions within the confined domains dynamically break‐reconstruct and nano‐effects impede the movement of the molecular chains. Macroscopically, this manifests itself as a hysteresis phenomenon present in every stretching cycle. However, after a relaxation period of 10 min, the samples exhibit a roughly recovery to their original state, indicating the self‐recovering property of the chain motion within the confining domains and the elastomer.

PDES‐ADM is believed to be capable of self‐healing at room temperature. A large number of nanoconfinement channels retain and reconfigure hydrogen bonding with the matrix when rupture occurs (Figure 1d). Supramolecular interactions on the surface and interior of the confined structural domains are believed to enable materials self‐healing properties. We placed the cut surfaces of the severed samples together and gently pressed them to heal at room temperature. The tensile strength, maximum strain, and toughness of PDES‐ADM after 24 h self‐healing reaches 94.22, 87.19, and 83.36% of the original samples (Figure 4e; Figure S7, Supporting Information). The samples were photographed before and after repair by an ultra‐deep 3D microscope system, and after 30 min of healing the scars had largely disappeared and were able to bend and twist normally and lift a bucket 36827 times their own weight (Figure 4f).^[^ ^42^, ^43^, ^44^, ^45^, ^46^, ^47^, ^48^, ^49^, ^50^, ^51^, ^52^, ^53^, ^54^, ^55^ ^]^

In order to validate the adaptability of material properties for practical sensing application scenarios, we compared recent years' flexible ion sensors identified as excellent performers. Our material has toughness and tensile strength on surpasses most of the flexible sensors for meeting the practical applications (Figure S16 Supporting Information). In addition, our results boast an excellent combination of performance, including toughness, adhesion, GF‐value functional reparability, and self‐healing capabilities (Figure 4g).

### 2.4 Strain‐Sensing Application

**Figure 5:**
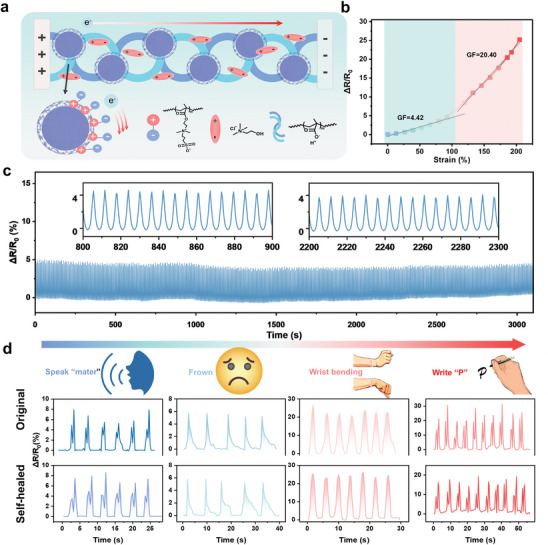
Strain sensing performance demonstration. a) Schematic diagram of the PDES‐ADM current sensing mechanism. Amphipathic ions make the confining domain a current channel. b) Relative resistance variation of PDES‐ADM as a function of tensile strain, and GF factor obtained by linear fitting. c) Reproducibility test for tensile cycles over 3000 s at 50% strain (Over 500 cycles). d) Signal outputs obtained from speech, facial expression, and action recognition of manufactured self‐healing sensors before and after self‐healing, respectively.

The dynamic nanoconfinement eutectogels have potential applications in the field of flexible sensors.^[^ ^56^ ^]^ PDES‐ADM possesses abundant amphipathic groups anchored on the surface and inside of the ADMs, which could generate abundant free ions to form electronic double layer with the electrodes in the presence of ChCl.^[^ ^57^ ^]^ As in **Figure** **5**a, based on the free ion directional migration ability provided by ChCl, electrons flow along ChCl and the surface of ADMs that have been oriented under an external electric field.^[^ ^58^ ^]^ Adhesion due to multiple internal hydrogen bonds enables the material to adhere stably to skin and various surfaces. We evaluated the adhesive properties using 2.25 cm^2^ samples for lap‐shear tests (Figure S13 Supporting Information). The adhesive strengths on two standard smooth surfaces are 399.354 Kpa (Metal) and 158.725 Kpa (PET), respectively. The excellent adhesion properties are sufficient to support stable adhesion on the skin surface. We monitored the resistive signals generated by the PDES‐ADM during various possible deformation motions by means of a Source meter with a digital multimeter.

A gauge factor (GF) ranging from 4.42 to 20.40 is obtained by calculating the slope of the resistance‐strain curve in tension (Figure 5b), which is sensitive enough to monitor most anthropogenic motions. This variation arises from disruptions in the conductive pathway by slippage of the polymer chains within the flexible sensor. According to the tunneling effect, the increased energy barrier at these breaks in the conductive pathway inhibits the transition of free electrons. Furthermore, the orientation of confined domains driven by stress significantly modifies the longitudinal structure of the eutectogel perpendicular to the conductive pathway, effectively resulting in an increase in fracture spacing and the splitting of the conductive pathway. This likewise triggered a decreasing trend in conductivity as shown in Figure S12 (Supporting Information). Cyclic tensile tests were conducted to verify the stability of the sensitivity (Figure 5c). The resistance changes spectra remain stable under 500 cycles within 3000 s, and there is a nearly unchanged signal output pattern under each band. Moreover, digital signal acquisition of several movements (including vocalization, facial and hand movements, etc.) was performed using the PDES‐ADM before and after healing (Figure 5d; Figure S15 Supporting Information). Both the original and repaired samples are able to maintain uniform and consistent signal outputs, indicating that a valid repair of ionic conductive pathways can be achieved by multiple hydrogen bonds and confined domains within the material. The resolved results of the resistance change spectra collected from the repaired samples also indicate that PDES‐ADM clearly reflects the individual motion signals (Figure S14 Supporting Information). The above results indicate that flexible sensing materials enhanced by amphiphilic ionization ADMs’ confinement effect have potential for further applications in wearable electronics.

## 3 Conclusion 

This study introduces a dynamic nanoconfinement approach that involves monomer trapping synthesis using amphiphilic sulfonated microspheres inspired by confinement in biological systems. A mechanical robust, low modulus, and self‐healing sensor is developed through this approach. Due to the strong internal hydrogen bonding and electrostatic interactions present, the confinement devices ADMs exhibit the ability to confine PDES at a volume exceeding 7000 times that of the original ADMs. The tightly interspersed molecular chains of PDES and ADMs match molecular chain motion scales of confined domains and substrate, which gives the flexible material a low modulus that matches the tissue motion. The presence of abundant sulfonic acid side groups facilitates the reestablishment of hydrogen bonding interactions between confined domains and the substrate post‐rupture, resulting in a reconfigurable effect distinct from conventional nanoconfined structures. Furthermore, the strain sensor composed of PDES‐ADM successfully monitored physiological activities by leveraging the ionization effect of sulfobetaine with ChCl. It is worth noting that the bulk nanoconfinement approach is not only limited to polymeric microsphere structures, but has yet to be applied to supramolecular assembly structures and mesoscopic microphase structures.

## 4 Experimental section

### Material

1‐Acryloylamino‐2‐methylpropanesulfonic acid (AMPS, 98%), 2‐(methacryloyloxy)ethyl]dimethyl‐(3‐sulphonatopropyl)ammonium hydroxide (DMAPS, 95%), 1‐hydroxycyclohexyl phenyl ketone (HCPK, 99%), cyclohexane (AR) and Span 80 (CR) were purchased from Shanghai Aladdin Biochemical Technology Company (China). N,N'‐Methylenebis(Acrylamide) (MBA, 99%) was purchased from Shanghai Saen Chemical Technology Company (China). Acrylic acid (AA, AR) and poly (ethylene glycol) diacrylate (PEG(200)‐DA) were purchased from Shanghai Macklin Biochemical Company (China). Choline chloride (ChCl) (99%) was purchased from Shanghai Adamas Reagent Company (China). 2‐Hydroxy‐4‐(2‐hydroxyethoxy)−2‐methylpropiophenone (photoinitiator 2959, ≥98%) was purchased from Shanghai Titan Technology Company (China). Tannic acid (TA, AR) was purchased from Sinopharm Pharmaceutical Control Pharmaceutical Chemical Reagent Company (China). The above drugs were used as received. The water used in all experiments was deionized and ultrafiltered to 18.2 MΩ cm using the Ulupure ultrapure water system. To perform the experiments on human subjects, rules or permissions from the relevant national or local authorities are not in place in the institution where the experiments were performed. Informed written consent was acquired from the human participants for the said experiments.

### Preparation of Dynamic Nanoconfinement Microspheres ADMs

In a spherical glass flask, DMAPS (0.97 mmol), AMPS (4.83 mmol), MBA (0.2 mmol), and HCPK (0.2 mmol) were added to 3 ml of water and stirred for 30 min, and Span80 (0.4 g) and cyclohexane (19.6 ml) were added and stirred for 30 min to form a homogeneous emulsion. The product was irradiated with UV light (60 w, 365 nm) for 5 min while stirring. The product obtained was centrifuged and washed twice with a cycle of cyclohexane, water, and isopropanol. The microsphere product ADMs were freeze‐dried and stored at room temperature.

### Preparation of AA/ChCl Deep Eutectic Solvent (DES)

Dry ChCl in a vacuum oven at 60 °C for 2 h before use. AA and ChCl were quickly added to a sealed flask in a 2:1 molar ratio with 0.1% TA small molecule as the conditioner. The solvent was stirred vigorously 60 °C for 2 h to obtain a colorless homogeneous solution. The obtained AA/ChCl type PDES was further stored in a vacuum desiccator filled with silica gel for further use.

### Preparation of PDES‐ADM Eutectogels

ADMs (0.015 g) and AA/ChCl type DES (5.906 g) were added to a sealed flask and slowly stirred at room temperature for 12 h to aid monomer infiltration. The monomer 0.1% photoinitiator 2959 was then added to the mixture and poured into a PTFE mold. DES was initiated for polymerization for 4 min using a UV dark box (8 w * 4, 360 nm). PDES and PDES‐PEGDA were prepared by following the same procedure without the addition of ADMs. Samples of monomer trapping synthesis at different times were also prepared in the same way.

### Characterization

A high vacuum field emission scanning electron microscope (JSM‐5600, JEOL, Japan) was used to observe the surface morphology of ADMs. FTIR spectral analysis of the ADMs materials was performed using a Nicolet iS50 FTIR spectrometer. The stress‐strain curves and shear adhesion strength of the elastomers were tested at room temperature using a universal material testing machine (Instron 5966, USA) with a tensile rate of 50 mm^−1^ min. The morphology of the ADMs nanoconfined microspheres was observed on a laser scanning confocal microscope (Zeiss LSM 710, Carl Zeiss, AG, Germany) and 3D cross‐section scanning images were reconstructed using 3D scanning mode. The sensing performance of the prepared electronic sensors was recorded at a constant voltage of 10 V using a Keithley 2601B source meter. The sensing performance of the prepared electronic sensors was recorded at a constant voltage of 10 V with a Keithley 2601B source meter. For human motion detection, the electronic sensors were mounted on the throat face, and hands of the volunteers to monitor the subtle movements of the muscles. Then, the source meter recorded the change of resistance signal in real time.

### Temperature‐Dependent FTIR Spectroscopy

The Nicolet iS50 Fourier transform spectrometer (USA) equipped with a homemade in situ pool (programmable heating device) was used for the temperature‐dependent FTIR experiments. The sample was sandwiched between two CaF_2_ windows and then heated at a heating rate of 5 °C min^−1^ from 30 to 180 °C. During the experiment, the sample was protected by high‐purity nitrogen gas (200 mL min^−1^). To obtain a high signal‐to‐noise ratio, all FTIR spectra were collected from 3800 to 900 cm^−1^ with a resolution of 4 cm^−1^ with 20 scans.

### SAXS Measurements

Scattering data were obtained with the SAXS instrument Xeuss 2.0 (France). A copper target phototube with a power of 30 W and a wavelength of 1.54189 Å was used. A Rayonix MX225‐HE CCD X‐ray detector was used with a sample‐to‐detector distance of 2480 mm. In situ tensile SAXS measurements were based on stretching in a small tensile machine, and SAXS data were recorded by the SAXS instrument at tensile strains of 0, 100, and 200%.

### Molecular Dynamics Simulation

Molecular dynamics simulations were conducted under the universal force field in Material Studio 2018. Initially, all molecules (including PDES, ADM, TA, PDES, PDES@ADM, and PDES@ADM@TA) were constructed using the Forcite module and geometrically optimized. After modeling each component, an overall system with minimized initial energy was built. To further optimize the conformations, isothermal‐isochoric molecular dynamics simulations were performed at 298 K for the PDES, PDES@ADMS, and PDES@ADM@TA composite material models. Subsequently, binding energy can be calculated based on the final conformation models.

## Conflict of Interest

The authors declare no conflict of interest.

## Supporting information



Supporting Information

## Data Availability

The data that support the findings of this study are available from the corresponding author upon reasonable request.
