# Supplementary material for: Monomer Trapping Synthesis Toward Dynamic Nanoconfinement Self‐healing Eutectogels for Strain Sensing
Source: Adv Sci (Weinh). 2024 Sep 16;11(42):2410446. doi: 10.1002/advs.202410446 (PMC11558160; doi:10.1002/advs.202410446)
Supplement: Supplementary file 1 — Supporting Information [file ADVS-11-2410446-s001.docx]

Supporting information for

**Monomer Trapping Synthesis towards Dynamic Nanoconfinement Self-healing Eutectogels for Strain Sensing**

*Yuesong Lv, Changchun Li, Zhangqin Yang, Mingxi Gan, Yuyan Wang, Minxun Lu ^*^, Xinxing Zhang ^*^ and Li Min*

Y. Lv, C. Li, Z. Yang, M. Gan, Prof. X. Zhang

State Key Laboratory of Polymer Materials Engineering, Polymer Research Institute of Sichuan University, Chengdu 610065, China.

E-mail: xxzwwh@scu.edu.cn

Dr. M. Lu, Dr. L. Min

Department of Orthopedics, Orthopedic Research Institute, Model Worker and Craftsman Talent Innovation Workshop of Sichuan Province, West China Hospital of Sichuan University, Chengdu, 610065, China.

E-mail: minhun@126.com

Y. Wang

Max Planck Institute for Polymer Research, Ackermannweg 10, Mainz 55128, Germany

**
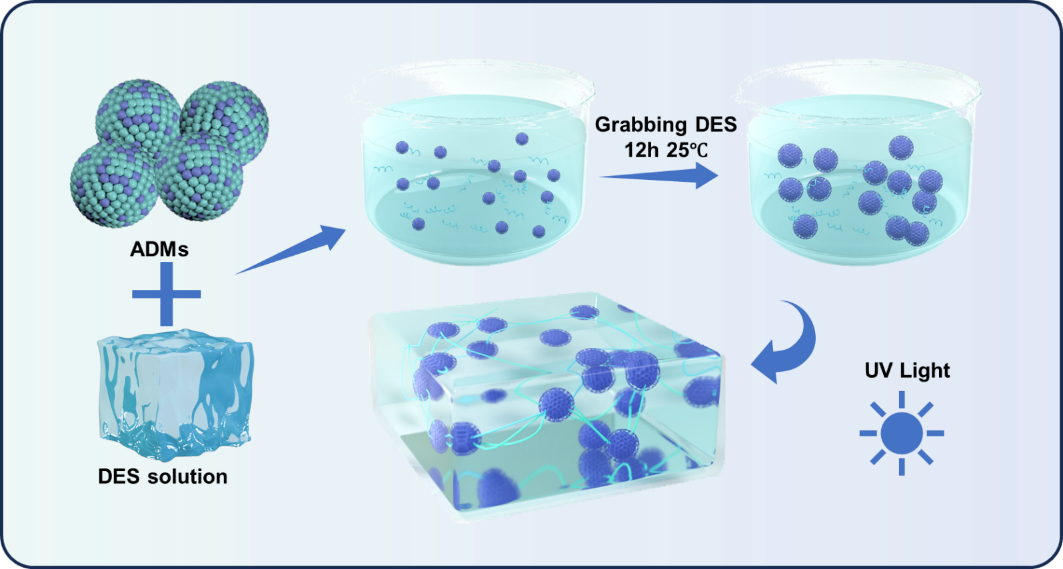
**

**Figure S1.** Preparation process of dynamic nanoconfinement Eutectogels.


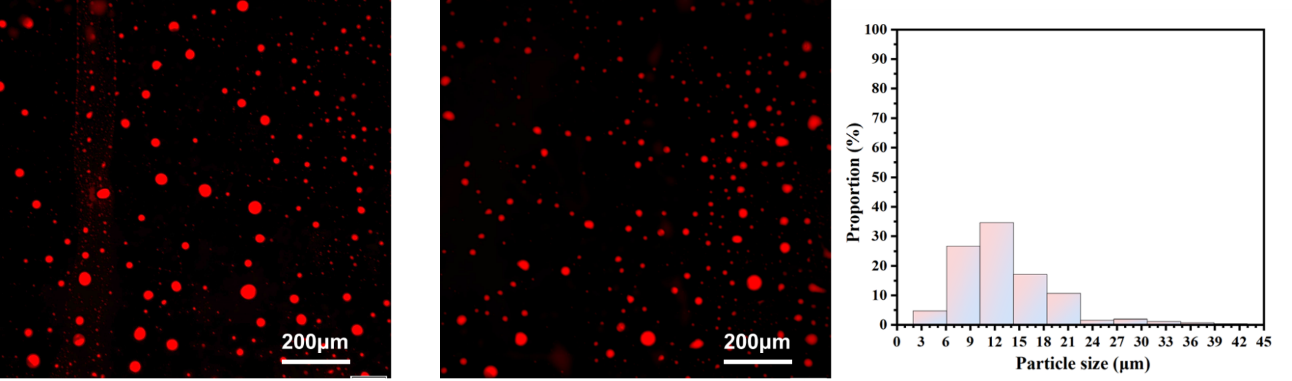


**Figure S2.** Laser confocal pictures of ADMs in isopropanol before swelling and their particle size distribution statistics.


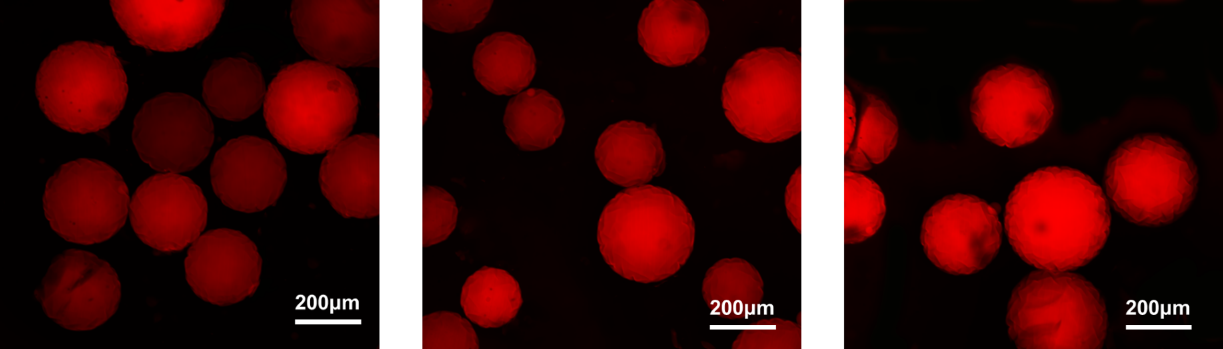


**Figure S3.** Laser confocal photographs of ADMs for counting post-solution particle size, obtained from three captured views at the same moment in time.


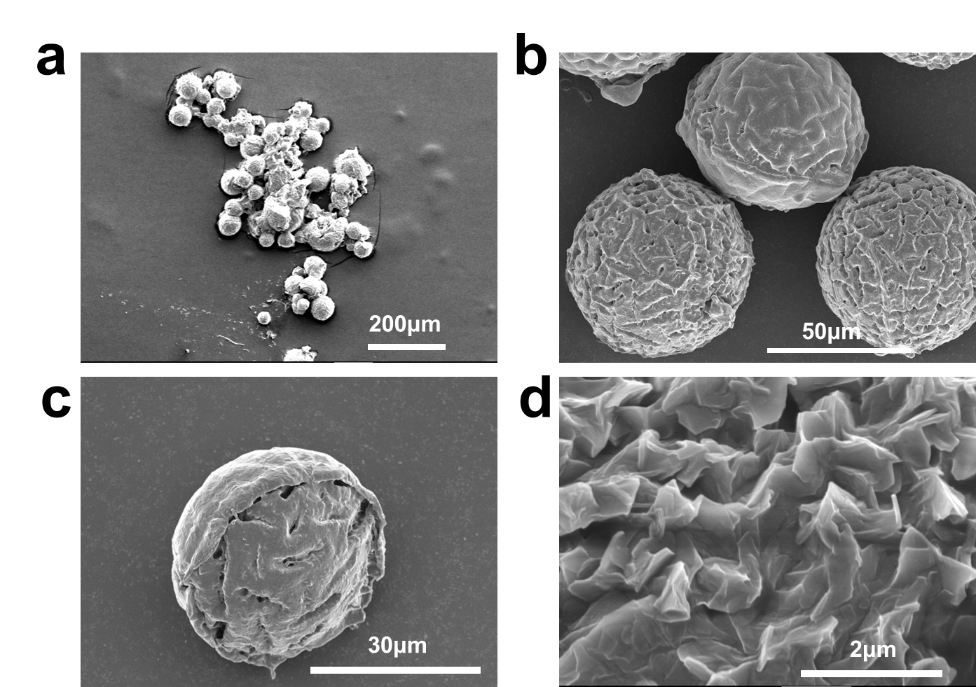


**Figure S4.** SEM images of ADMs treated by partial swelling followed by freeze-drying, respectively (a) aggregated morphology, (b) partial magnification, (c) fractured microspheres, (d) cross-section magnification.


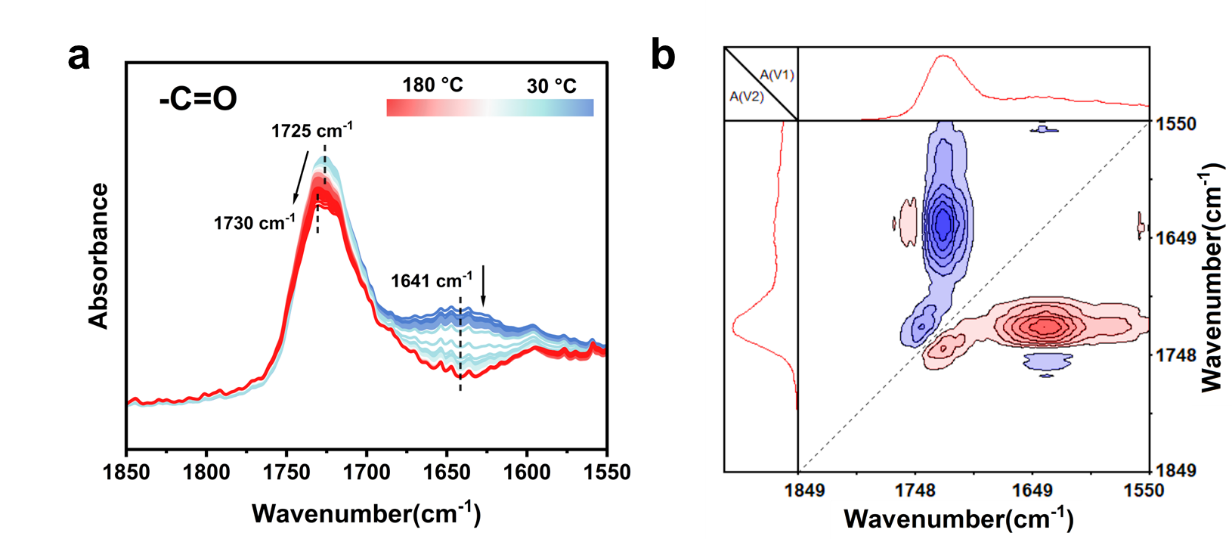


**Figure S5.** a) Temperature-variable FTIR spectra of the PDES-ADM upon heating from 30 to 180 °C in the range of 1850–1550. b) Generalized 2D correlation spectra of the PDES-ADM upon heating from 30 to 180 °C in the range of 1850–1550.


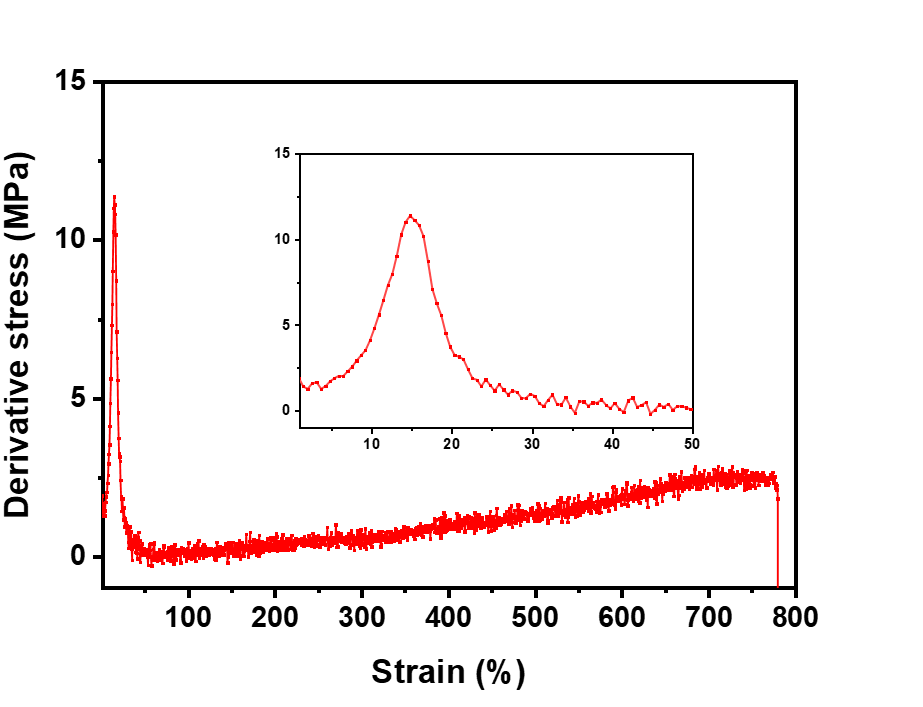


**Figure S6.** First-order differential curve of the stress-strain curve of PDES-ADM, inset is zoomed in to the 50% strain range.


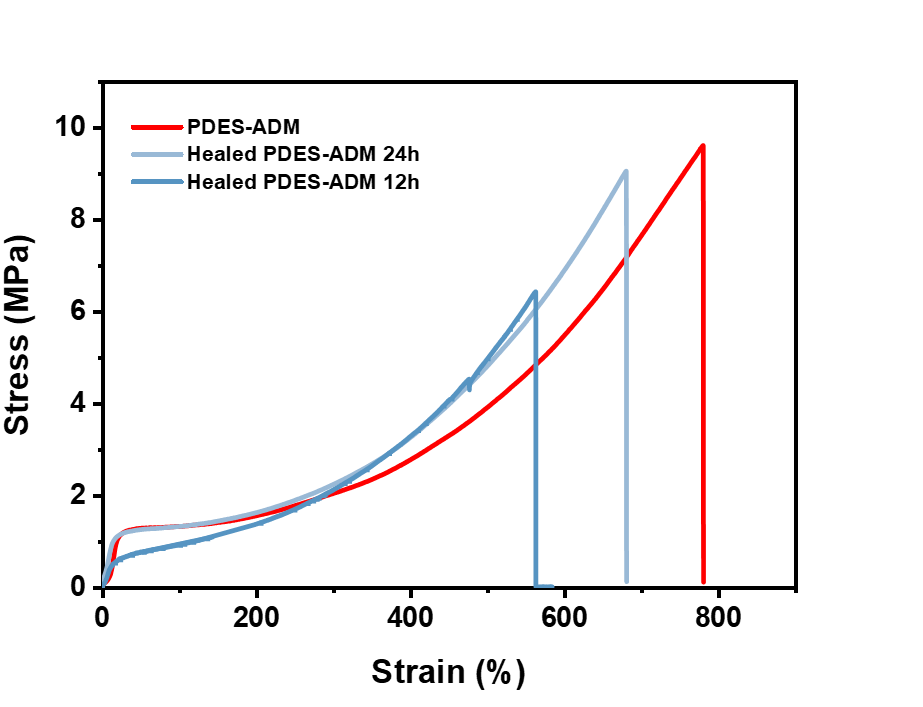


**Figure S7.** Stress-strain curves of original PDES-ADM and after 12h or 24h of room temperature self-healing.

**Figure S8.** Stress-strain curves of eutectogels obtained by controlling different capture times during monomer trapping polymerization.


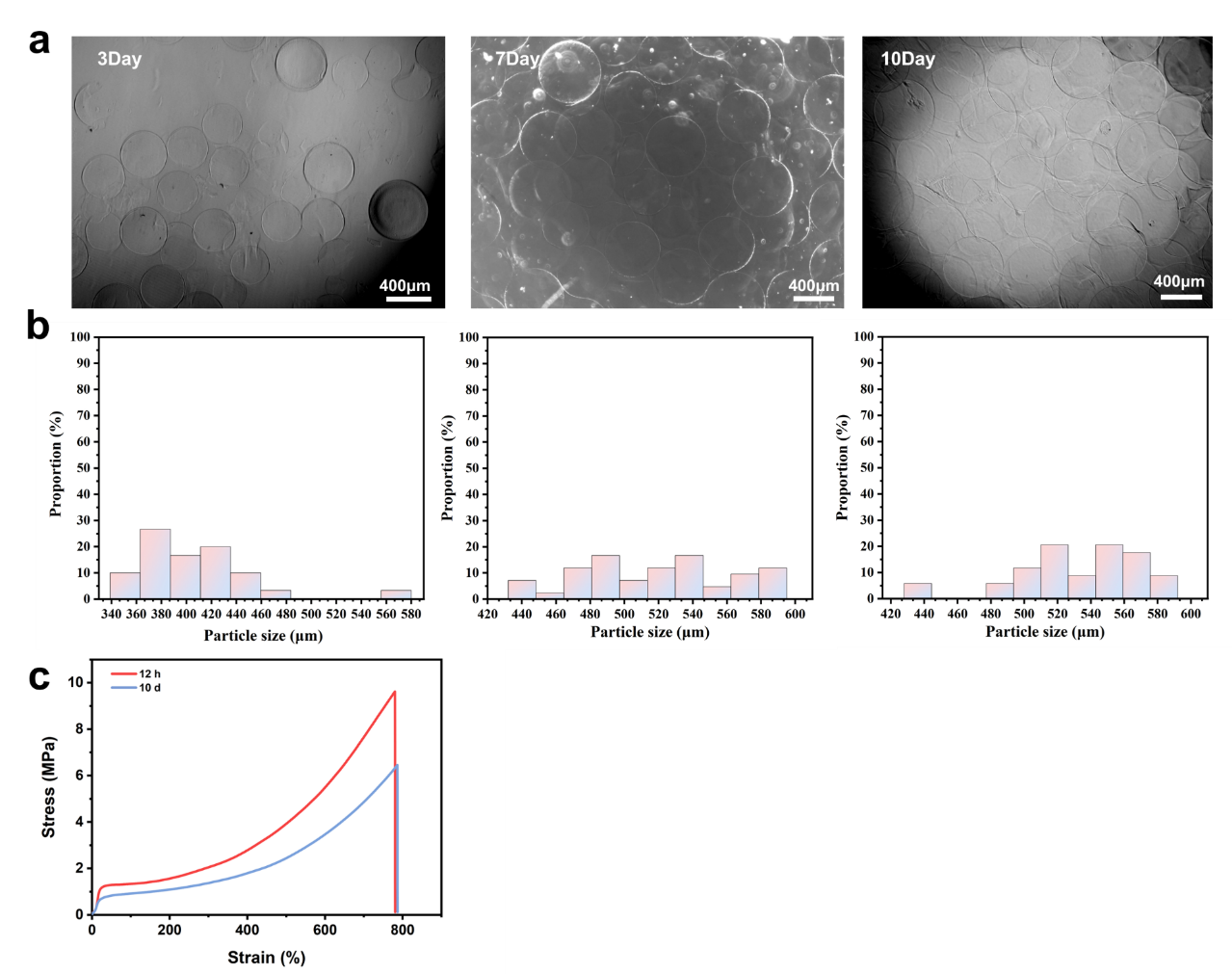


**Figure S9.** a) Morphological pictures of ADM and it mean sizes (420.56 μm at 3 days, 517.45 μm at 7 days and 532.72 μm at 10 days). b) The particle size distribution after different days of monomer trapping. c) Stress-strain curves of eutectogels obtained by polymerisation after 10 days of monomer trapping.


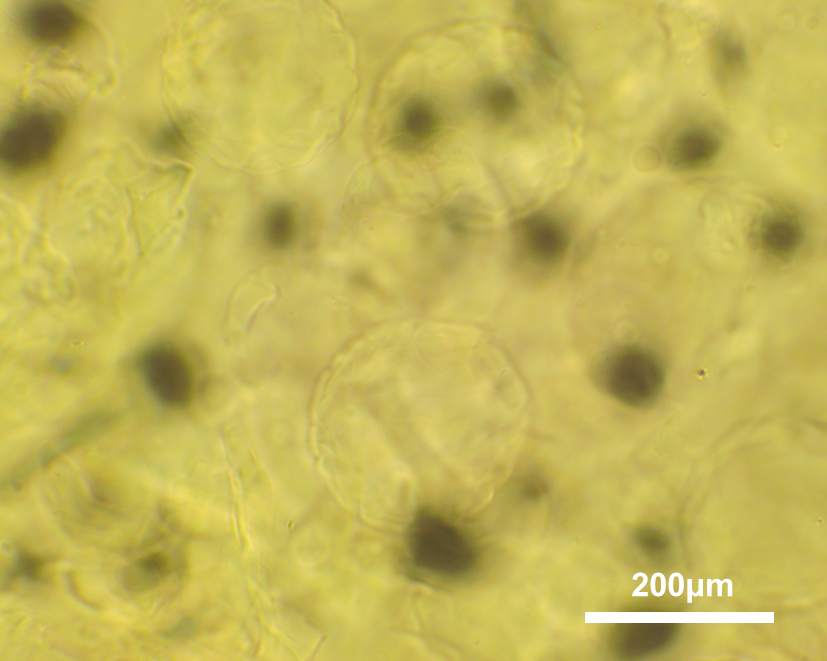


**Figure S10.** Optical photographs of the confined domains in PDES-ADM in dark field taken by optical microscopy show different light refraction effects due to the sparsely distributed ADM skeleton inside.


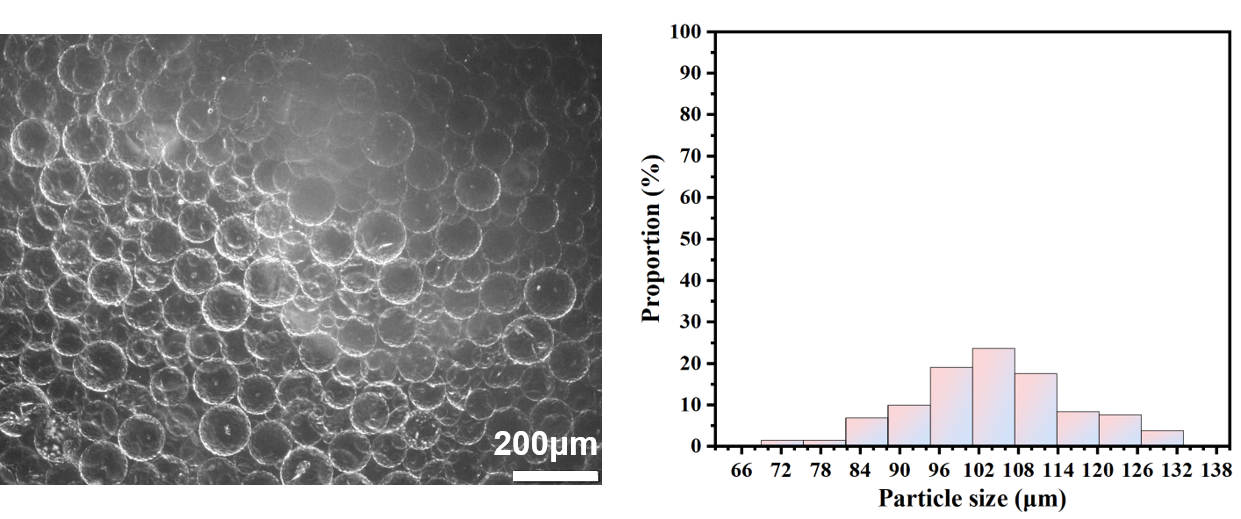


**Figure S11.** Morphology and particle size distribution of ADM undergoing 12h monomer trapping without tannic acid assistance.


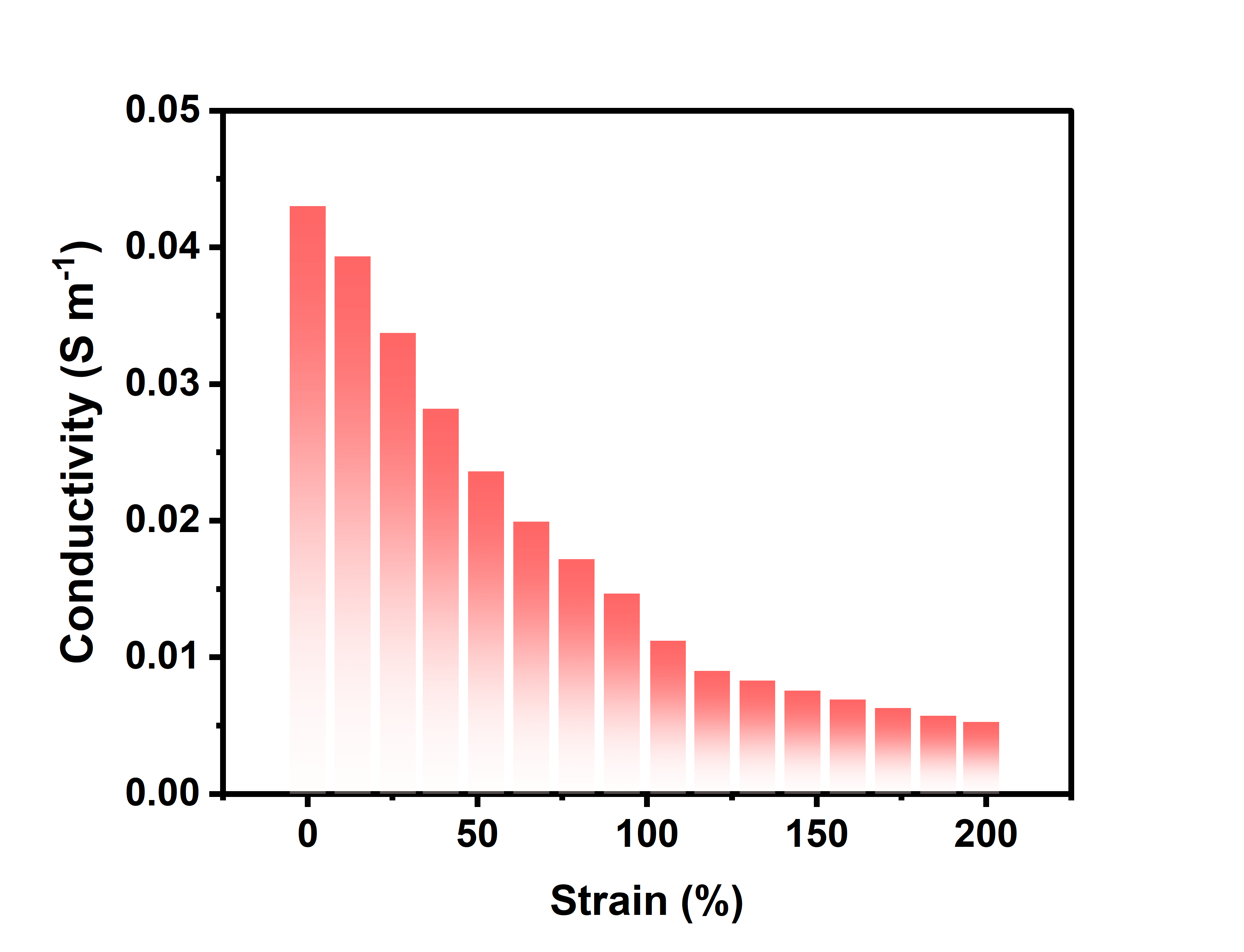


**Figure S12**. The conductivity variation of PDES-ADM in stretch overcharge. Calculated by measuring the in situ alternating resistance according to σ = Gl/A, where G is the conductance, A is the cross-sectional area of the conductor, and I is the length of the conductor. (Assuming linear reduction of the cross-sectional area during stretching)


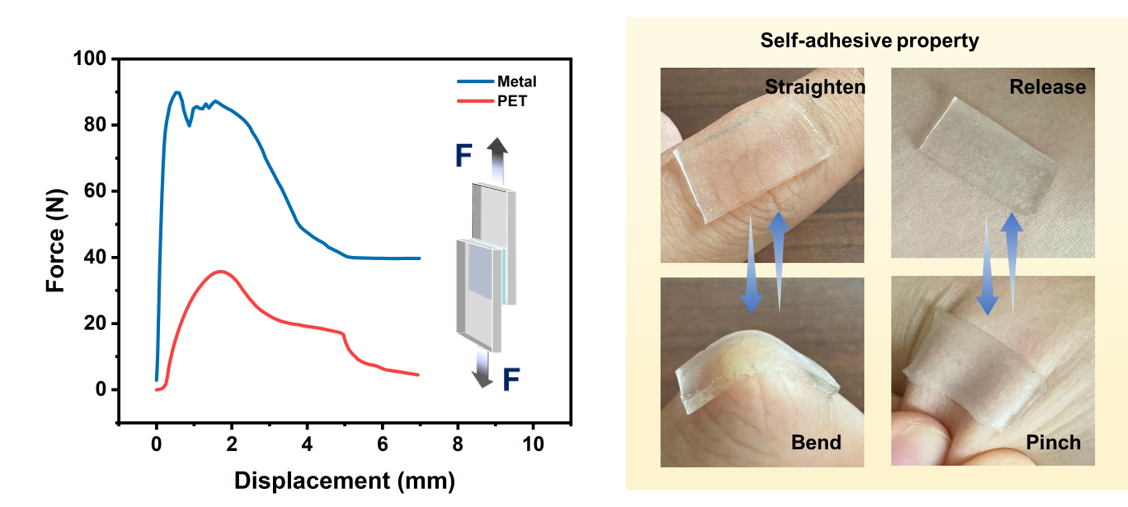


**Figure S13**. Shear adhesion profile of PDES-ADM on smooth metal and plastic surfaces. And its optical picture of the movement with skin


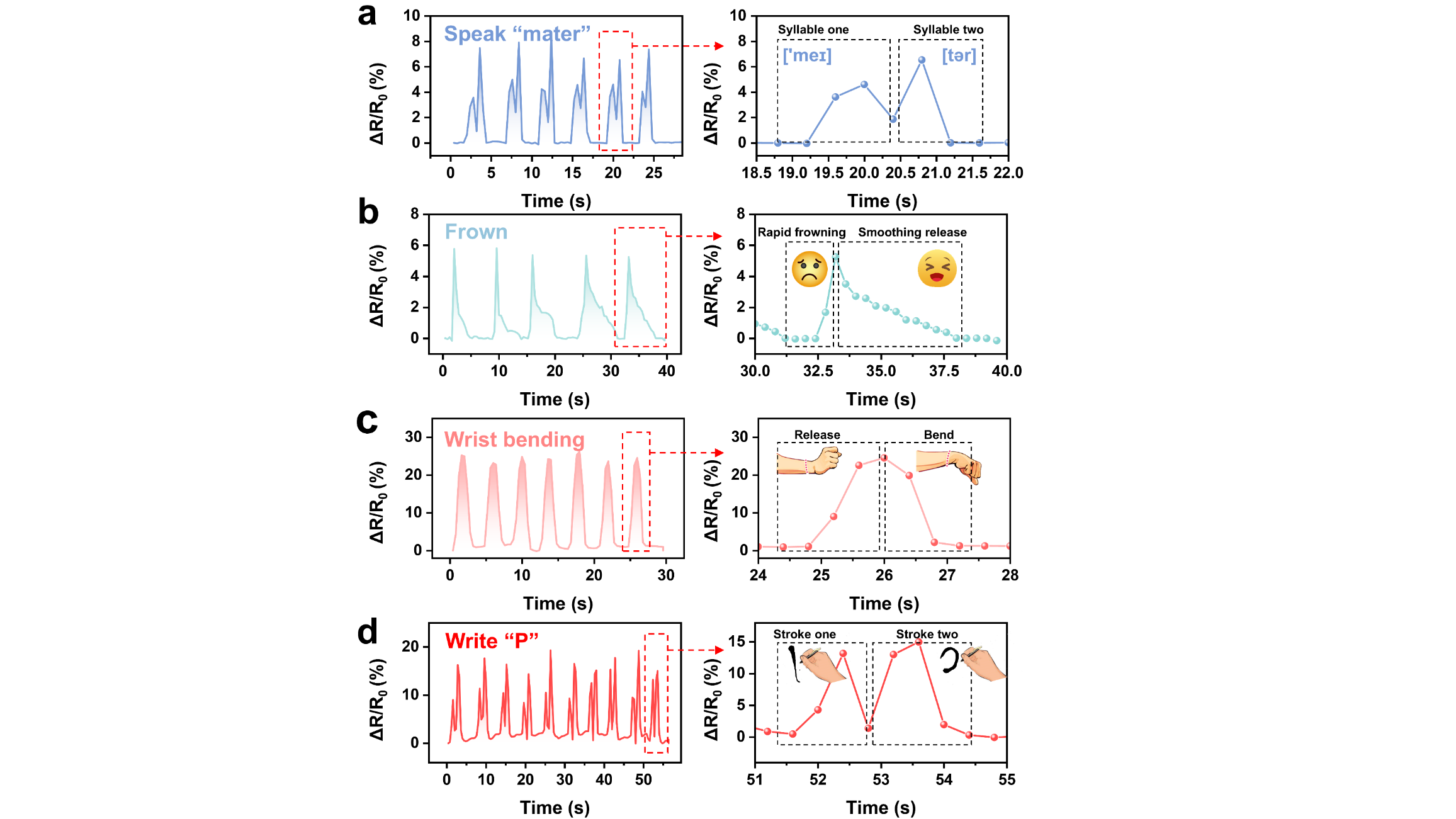


**Figure S14.** (a) Eutectic gel detects weak current changes due to different glottal twitching of drawn-out ['meɪ] versus crisp [tər] when the two syllables of “Mater.” are spoken aloud. (b) During facial expression “frowning”, the eutectic gel detects a fast-climbing peak and a slow falling peak of the electrical signal triggered by rapid frowning and slow stretching of the facial expression, respectively. (c) The up and down bending activity of the wrist induces a small elongation and restitution of the eutectic gel, which exhibits a single peak. (d) During the writing of P there will be two different rotations of the wrist during the two strokes, with the second stroke being slightly larger than the first. The eutectic gel can sensitively capture the change in deviation amplitude, thus producing two signal peaks that are closely adjacent to each other.


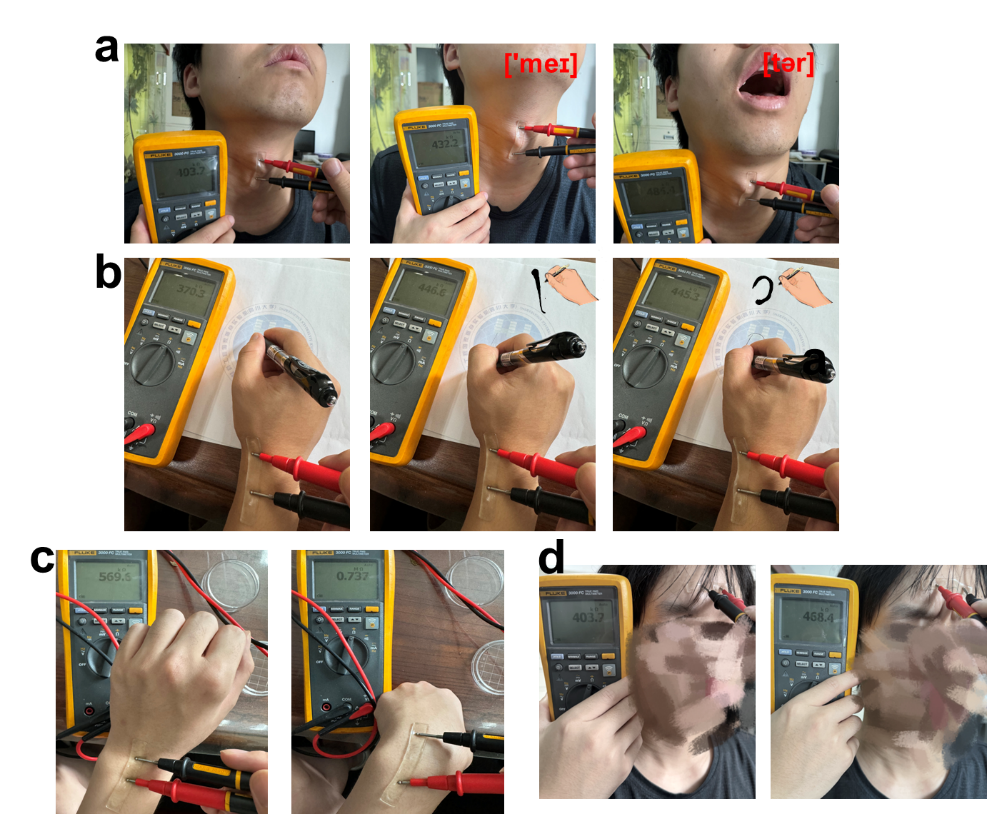


**Figure S15.** Photographs of the four actions for signal acquisition, corresponding to a) Speak ‘mater’, b) Write ‘P’, c) Wrist bending, and d) Frown.


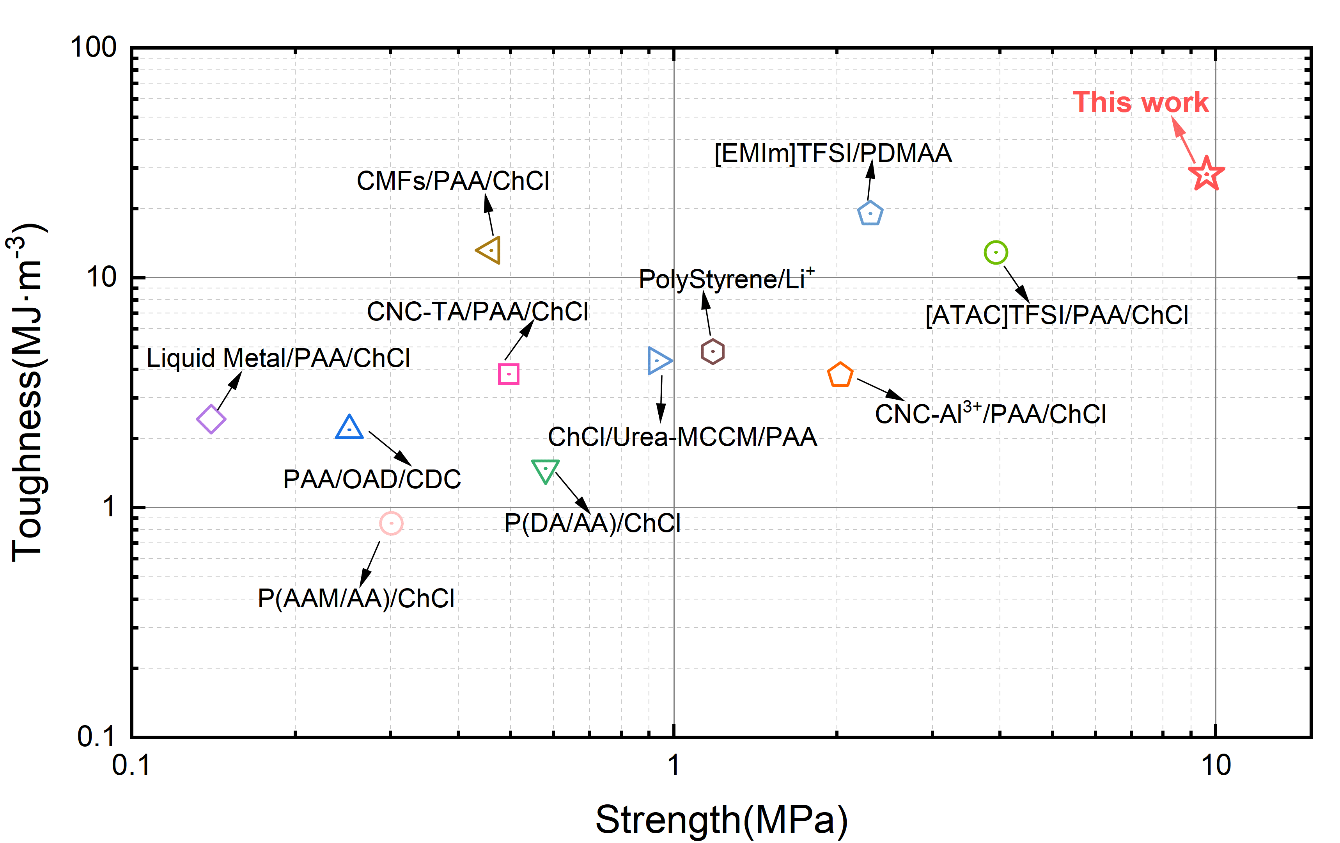


**Figure S16.** Comparison of toughness and tensile strength of PDES-ADM with flexible ion sensors of recent years.

**Table S1. Performance comparison of PDES-ADM with recently reported tough flexible ion sensors.**

| **Materials** | **Strength**  **(Mpa)** | **Toughness**  **(MJ/m^3^)** | **Self-healing efficiency** | | **Adhesion to Skin** | **GF values** | **Electrical self-healing** | **References** |
| --- | --- | --- | --- | --- | --- | --- | --- | --- |
| P(AAM/AA)/ChCl | 0.301 | 0.855 | | 91% | no | nnone | yes | “Autonomous Self-Healing, Antifreezing, and Transparent Conductive Elastomers.” ***Chem. Mater.*** (2020), (32) |
| PAA/OAD/CDC | 0.252 | 2.175 | | 92% | yes | none | yes | “Self-Adhesive Dry Ionic Conductors Based on Supramolecular Deep Eutectic Polymers.” ***Chem. Mater.*** (2022), (34) |
| P(DA/AA)/ChCl | 0.58 | 1.48 | | 90% | yes | none | yes | “A Repeatable Self-Adhesive Liquid-Free Double-Network Ionic Conductor with Tunable Multifunctionality.” ***ACS Appl. Mater. Interfaces*** (2022), (14) |
| Liquid Metal/PAA/ChCl | 0.14 | 2.43 | | 98% | yes | 2.17 | yes | “Multifunctional Liquid-Free Ionic Conductive Elastomer Fabricated by Liquid Metal Induced Polymerization.” ***Adv. Funct. Mater.*** (2021), (31) |
| CMFs/PAA/ChCl | 0.46 | 13.17 | | 85% | no | 3.71 | no | “Tough and Ultrastretchable Liquid-Free Ion Conductor Strengthened by Deep Eutectic Solvent Hydrolyzed Cellulose Microfibers.” ***Adv. Funct. Mater.*** (2022), (32) |
| ChCl/Urea-MCCM-AA | 0.93 | 4.36 | | None | no | 1.222 | no | “Liquid-Free, Anti-Freezing, Solvent-Resistant, Cellulose-Derived Ionic Conductive Elastomer for Stretchable Wearable Electronics and Triboelectric Nanogenerators.” ***Adv. Funct. Mater.*** (2022), (32) |
| PolyStyrene/Li+ | 1.18 | 4.779 | | 95.76% | no | 1.45 | yes | “Liquid-free ionic conductive elastomers with high mechanical properties and ionic conductivity for multifunctional sensors and triboelectric nanogenerators.” ***Mater. Horiz.*** (2024), (**11)** |
| CNC-TA/PAA/ChCl | 0.497 | 3.81 | | 53.10% | yes | 3.67 | yes | “Tough Liquid-Free Ionic Conductive Elastomers with Robust Adhesion and Self-Healing Properties for Ionotronic Devices.” ***Adv. Funct. Mater.*** (2024), (34) |
| CNC-Al^3+^/PAA/ChCl | 2.03 | 3.77 | | None | no | 1.06 | no | “Liquid-free, tough and transparent ionic conductive elastomers based on nanocellulose for multi-functional sensors and triboelectric nanogenerators.” [***Nano Energy***](https://www.sciencedirect.com/journal/nano-energy) (2024), (129) |
| [EMIm]TFSI/PDMAA | 2.306 | 19 | | None | no | 9.8 | no | “Recyclable, Healable, and Tough Ionogels Insensitive to Crack Propagation.” ***Adv. Mater.*** (2022), (34) |
| [ATAC][TFSI]/PAA/ChCl | 3.93 | 12.89 | | 78.90% | yes | 2.5 | no | “Recyclable multifunctional ion conductive elastomers for strain/temperature sensors and bioelectrodes.” ***Chem. Eng. J.*** (2024), (489) |
| **This work** | **9.6178** | **28.18** | | **94.22%** | **yes** | **20.4** | **yes** | **This work** |

**References**

[1] R. Li, T. Fan, G. Chen, K. Zhang, B. Su, J. Tian, M. He, *Chem. Mater.* **2020**, *32*, 874.

[2] K. Zhang, R. Li, G. Chen, X. Wang, M. He, *Chem. Mater.* **2022**, *34*, 3736.

[3] Z. Hua, G. Chen, K. Zhao, R. Li, M. He, *ACS Appl. Mater. Interfaces* **2022**, *14*, 22418.

[4] M. Wang, Z. Lai, X. Jin, T. Sun, H. Liu, H. Qi, *Adv. Funct. Mater.* **2021**, *31*, 2101957.

[5] X. Sun, Y. Zhu, J. Zhu, K. Le, P. Servati, F. Jiang, *Adv. Funct. Mater.* **2022**, *32*, 2202533.

[6] C. Lu, X. Wang, Y. Shen, C. Wang, J. Wang, Q. Yong, F. Chu, *Adv. Funct. Mater.* **2022**, *32*, 2207714.

[7] F. Ou, T. Xie, X. Li, Z. Zhang, C. Ning, L. Tuo, W. Pan, C. Wang, X. Duan, Q. Liang, W. Gao, Z. Li, S. Zhao, *Mater. Horiz.* **2024**, *11*, 2191.

[8] X. Zhang, Q. Fu, Y. Wang, H. Zhao, S. Hao, C. Ma, F. Xu, J. Yang, *Adv. Funct. Mater.* **2024**, *34*, 2307400.

[9] Y. Lan, W. Liu, Z. Lv, Z. Li, A. Dufresne, L. Fu, B. Lin, C. Xu, B. Huang, *Nano Energy* **2024**, *129*, 110047.

[10] W. Li, L. Li, S. Zheng, Z. Liu, X. Zou, Z. Sun, J. Guo, F. Yan, *Adv. Mater.* **2022**, *34*, 2203049.

[11] C. Zhou, X. Song, W. Xia, S. Liu, Z. Wu, H. Chen, *Chem. Eng. J.* **2024**, *489*, 151433.
